# MTHFR 1298C and 677T Interactions with APOE e4 and White Matter Hyperintensity Volume on Amyloid‐Positive Status in Cognitively Unimpaired Older Adults

**DOI:** 10.1002/alz70856_097655

**Published:** 2025-12-24

**Authors:** Dillan Patel, Michael H. Malek‐Ahmadi, Brandon Pitts, Ignazio Piras, Kewei Chen, Vivek Devadas, Ji Luo, Yi Su

**Affiliations:** ^1^ Rice University, Houston, TX, USA; ^2^ Banner Alzheimer's Institute, Phoenix, AZ, USA; ^3^ Arizona Alzheimer's Consortium, Phoenix, AZ, USA; ^4^ University of Arizona College of Medicine‐Phoenix, Phoenix, AZ, USA; ^5^ Marquette University, Milwaukee, WI, USA; ^6^ The Translational Genomics Research Institute (TGen‐ an Affiliate of City of Hope), Phoenix, AZ, USA; ^7^ School of Mathematics and Statistical Sciences, College of Health Solutions, Arizona State University, Tempe, AZ, USA; ^8^ Arizona State University, Tempe, AZ, USA; ^9^ University of Arizona College of Medicine Phoenix, Phoenix, AZ, USA; ^10^ ASU‐Mayo Center for Innovative Imaging, Tempe, AZ, USA

## Abstract

**Background:**

The 677T and 1298C alleles of MTHFR (methylenetetrahydrofolate reductase) are both involved in homocysteine processing and are associated with increased risks for both Alzheimer's disease (AD) and cardiovascular disease. Given the role of vascular factors in AD, we investigated whether MTHFR allele interactions with APOE e4 and white matter hyperintensity volume (WMHV) are associated with amyloid‐positivity in cognitively unimpaired (CU) older adults.

**Method:**

Data from 341 CU subjects (Age: 74.34±6.94, 58% Female, 32% e4 carriers, 33% amyloid‐positive) from the Alzheimer's Disease Neuroimaging Initiative (ADNI) were analyzed. Amyloid‐PET SUVR of >=1.11 was used to classify amyloid positivity. WMHV was quantified using the Lesion Segmentation Toolbox. Separate logistic regression models that adjusted for age and sex were used to investigate the contribution of the MTHFR 1298C and MTHFR 677T alleles individually as well as their interactions with APOE e4 carrier status and with WMHV on amyloid positivity.

**Result:**

Main effects for both MTHFR alleles were not significant (1298C: OR = 1.45, 95% CI = (0.56, 3.74), *p* =  0.44); (677T: OR = 0.59, 95% CI = (0.23, 1.54), *p* =  0.28). 1298C interactions with APOE e4 carrier status (OR = 0.35, 95% CI = (0.08, 1.43), *p* =  0.14) and WMHV (OR = 0.99, 95% CI = (0.90, 1.08), *p* =  0.84) were not associated with amyloid positivity. There was a significant interaction for 677T and APOE e4 carrier status (OR = 4.53, 95% CI = (1.01, 19.69), *p* =  0.04), but not with WMHV (OR = 1.08, 95% CI = (0.99, 1.19), *p* =  0.06).

**Conclusion:**

CU individuals who carry the APOE e4 and 677T allele of MTHFR are more likely to be classified as amyloid‐positive. Treatments that target vascular‐related APOE e4 and MTHFR 677T pathways may help ameliorate downstream amyloid pathology in AD.

